# Sequencing of Linkage Region on Chromosome 12p11 Identifies *PKP2* as a Candidate Gene for Left Ventricular Mass in Dominican Families

**DOI:** 10.1534/g3.117.300358

**Published:** 2017-12-29

**Authors:** Nicole D. Dueker, Shengru Guo, Ashley Beecham, Liyong Wang, Susan H. Blanton, Marco R. Di Tullio, Tatjana Rundek, Ralph L. Sacco

**Affiliations:** * John P. Hussman Institute for Human Genomics, University of Miami, Florida 33136; † Dr. John T. Macdonald Foundation Department of Human Genetics, University of Miami, Florida 33136; ‡ Department of Medicine, Columbia University, New York, New York 10032; § Department of Neurology, Miller School of Medicine, University of Miami, Florida 33136; ** Department of Public Health Sciences, Miller School of Medicine, University of Miami, Florida 33136

**Keywords:** rare variants, DNA sequencing, left ventricle, plakophilin, Hispanics

## Abstract

Increased left ventricular mass (LVM) is an intermediate phenotype for cardiovascular disease (CVD) and a predictor of stroke. Using families from the Dominican Republic, we have previously shown LVM to be heritable and found evidence for linkage to chromosome 12p11. Our current study aimed to further characterize the QTL by sequencing the 1 LOD unit down region in 10 families from the Dominican Republic with evidence for linkage to LVM. Within this region, we tested 5477 common variants [CVs; minor allele frequency (MAF) ≥5%] using the Quantitative Transmission-Disequilibrium Test (QTDT). Gene-based analyses were performed to test rare variants (RVs; MAF < 5%) in 181 genes using the family-based sequence kernel association test. A sample of 618 unrelated Dominicans from the Northern Manhattan Study (NOMAS) and 12 Dominican families with Exome Array data were used for replication analyses. The most strongly associated CV with evidence for replication was rs1046116 (Discovery families *P* = 9.0 × 10^−4^; NOMAS *P* = 0.03; replication families *P* = 0.46), a missense variant in *PKP2*. In nonsynonymous RV analyses, *PKP2* was one of the most strongly associated genes (*P* = 0.05) with suggestive evidence for replication in NOMAS (*P* = 0.05). *PKP2* encodes the plakophilin 2 protein and is a desmosomal gene implicated in arrythmogenic right ventricular cardiomyopathy and recently in arrhythmogenic left ventricular cardiomyopathy, which makes *PKP*2 an excellent candidate gene for LVM. In conclusion, sequencing of our previously reported QTL identified common and rare variants within *PKP2* to be associated with LVM. Future studies are necessary to elucidate the role these variants play in influencing LVM.

Cardiovascular disease (CVD) is a significant public health burden, affecting more than one in three American adults ≥20 yr old and resulting in an estimated $316.6 billion in medical costs in 2012 (Mozaffarian 2016). Increased left ventricular mass (LVM), an intermediate phenotype for CVD, is predictive of stroke and CVD events (Levy *et al.* 1990; Devereux *et al.* 2004; Rodriguez *et al.* 2006). Traditional factors, including elevated blood pressure, body mass index, and weight, are known to influence LVM (Savage *et al.* 1990; Garner *et al.* 2000). Heritability studies suggest that LVM may be genetically controlled, with reported heritability estimates ranging from 0.24 to 0.70 in various populations (Post *et al.* 1997; Swan *et al.* 2003; Juo *et al.* 2005; Sharma *et al.* 2006; Assimes *et al.* 2007; de Simone *et al.* 2007; Wang *et al.* 2009). In addition, genome-wide association studies, primarily performed in samples of European ancestry, have identified significant associations between LVM and common variants within several loci (Vasan *et al.* 2009; Fox *et al.* 2013). A large meta-analysis of individuals of European ancestry found variants within 14q12 and 2p21 to be associated with decreased LVM and variants within 15q14 to be associated with increased LVM (Vasan *et al.* 2009). In a meta-analysis of African American cohorts, variants within 8q11 were associated with increased LVM (Fox *et al.* 2013). Though these loci were significantly associated with LVM in the discovery cohorts of each study, none achieved replication in their respective validation cohorts. Additional variants have been implicated in LVM through other genome-wide association studies, although none achieved statistical significance (Vasan *et al.* 2007; Arnett *et al.* 2009; Harper *et al.* 2013).

In a previous genome-wide linkage study, we identified a novel region on chromosome 12p11 linked to LVM using extended families from the Dominican Republic (MLOD = 3.11), particularly in families with higher waist circumference (MLOD = 4.45) (Wang *et al.* 2009). We further characterized this region by investigating common variants in a population-based cohort of Caribbean Hispanics and identified several candidates, including *SOX5* (Della-Morte *et al.* 2011). For this study we aimed to identify additional common and rare variants contributing to the 12p11 linkage signal by sequencing the 1 LOD down region on 12p11 in 10 extended Dominican families with strong evidence for linkage to this region (LOD ≥ 0.1). We validated the observed associations using both genome-wide and exome-wide genotype data from unrelated Dominicans in the Northern Manhattan Study (NOMAS) as well as exome-wide genotype data from 12 additional extended Dominican families with suggestive evidence for linkage to the 12p11 region.

## Materials and Methods

### Study samples

Individuals from both NOMAS, a population-based cohort, and the Family Study of Stroke Risk and Carotid Atherosclerosis, a family study consisting of select probands from NOMAS and their family members (Sacco *et al.* 2004), were used for the current study. Details of NOMAS and the Family Study have been published previously (Sacco *et al.* 2004, 2007). Briefly, a total of 3298 stroke-free population-based participants were enrolled in NOMAS from 1993 to 2001. The family study enrolled a subset of Caribbean Hispanic probands from NOMAS with a high risk of cardiovascular disease (Sacco *et al.* 2007) who could provide a family history, obtain family members’ permission for research staff to contact them, and have at least three first-degree relatives able to participate. All probands were identified in northern Manhattan and family members were enrolled in New York at Columbia University and in the Dominican Republic (DR) at the Clinicas Corazones Unidos in Santo Domingo. All participants provided written informed consent and the study was approved by the Institutional Review Boards of Columbia University, University of Miami, the National Bioethics Committee, and the Independent Ethics Committee of Instituto Oncologico Regional del Cibao in the DR (20070478 and 20072012).

Twenty-two families with a family-specific LOD score >0.1 at the chromosome 12p11 QTL for LVM were selected for our current study; 10 families were sequenced as part of our discovery analyses (*n* = 180 individuals) (Table 1) and 12 families were genotyped on the Exome Array for our replication analyses (*n* = 143 individuals) (Supplemental Material, Table S1). Details of the family selection have been published previously (Dueker *et al.* 2016). Additional replication analyses were performed using a sample of 618 unrelated Dominicans from NOMAS (Sacco *et al.* 2004) who were not enrolled in the family study.

**Table 1:** Characteristics of Dominican families included in analyses for chr12p11 analyses

Family ID	Individuals per Family	Family-Specific LOD Score	LV Mass Residual (g)				Age (yr) µ ± SD	BMI (kg/m^2^) µ ± SD	Waist Circumference (inch) µ ± SD	% Female	% Diabetes	% Smoker	% Hypertension
			Mean	SD	Min	Max							
5275	24	0.94	−0.10	2.71	−4.77	6.16	44.0 ± 15.7	33.3	31.2 ± 6.3	38.0 ± 6.2	12.5	33.3	33.3
3719	12	0.92	0.43	2.64	−3.04	5.36	49.3 ± 16.2	33.3	32.3 ± 7.8	37.8 ± 5.4	8.3	33.3	41.7
5103	35	0.44	1.31	2.34	−3.30	6.89	42.2 ± 17.8	37.1	26.9 ± 5.3	36.3 ± 5.8	9.6	37.1	42.9
2235	15	0.37	−0.46	3.40	−5.62	5.54	49.3 ± 16.9	60.0	29.1 ± 3.4	39.0 ± 5.6	20.0	60.0	66.7
4641	13	0.30	−0.44	2.44	−4.37	4.04	42.9 ± 18.0	53.8	31.3 ± 8.5	37.3 ± 6.1	53.8	53.8	23.1
2783	15	0.21	−0.57	3.00	−3.97	6.66	40.7 ± 15.4	26.7	29.5 ± 6.0	36.9 ± 5.2	13.3	26.7	53.3
6081	27	0.20	−0.55	1.45	−3.13	2.47	46.3 ± 16.9	14.8	27.7 ± 5.4	36.0 ± 5.3	3.7	14.8	40.7
3561	17	0.16	−0.06	3.18	−4.99	6.70	47.1 ± 13.8	41.2	35.3 ± 7.5	42.2 ± 6.9	29.4	41.2	52.9
1917	10	0.15	0.20	2.84	−4.06	4.01	45.8 ± 16.8	80.0	33.8 ± 7.2	41.0 ± 6.0	20.0	80.0	60.0
803	12	0.14	−1.16	2.10	−4.25	3.11	54.7 ± 24.2	41.7	30.3 ± 5.2	38.8 ± 5.1	16.7	41.7	41.7

### Echocardiographic evaluation and risk factor measurements

As detailed previously (Wang *et al.* 2009; Della-Morte *et al.* 2011), standard two-dimensional echocardiography, including color-Doppler flow study was performed according to the guidelines of the American Society of Echocardiography (Sahn *et al.* 1978). High quality parasternal long axis views of the left ventricle were obtained, from which left ventricular end-diastolic diameter (LVDD), left ventricular end-systolic diameter (LVSD), interventricular septum (IVS), and posterior wall thickness (PWT) were derived (Di Tullio *et al.* 2003). LVM was calculated according to the modified American Society of Echocardiography formula: LVM = 0.8 [1.04 (LVDD + IVS + PWT)^3^ − (LVDD)^3^] + 0.6 (Devereux *et al.* 1986).

Vascular risk factors, including body mass index (BMI), systolic blood pressure, and smoking status, were collected during a standardized interview (Elkind *et al.* 2006). Smoking status was defined as never *vs.* ever, systolic blood pressure was defined as the average of two separate systolic blood pressure measurements taken after rest, and presence of diabetes was defined as fasting blood glucose level ≥126 mg/dl or self-reported history of diabetes. All data collection procedures were standardized and identical across NOMAS and the Family Study.

### Discovery sample sequencing and quality control

In the family sample, genomic DNA was isolated from whole blood. Targeted sequencing of the exons in 181 genes within the 1 LOD unit down region on 12p11 (chr12:24–51 Mb), as well as sequencing beyond the exons for candidate genes identified previously (*ARID2*, *BICD1*, *BIN2*, *c12orf68*, *FAR2*, *RACGAP1*, *SLC38A1*, *SOX5*) (Della-Morte *et al.* 2011), was performed using a customized Agilent SureSelect Enrichment kit. A detailed description of sequencing methods has been previously published (Wang *et al.* 2015). Briefly, DNA libraries were sequenced on an Illumina HiSeq2000 and the raw sequencing reads were aligned to the human reference sequence hg19 with the Burrows-Wheeler Aligner (Li and Durbin 2010). Variant calling was performed using the Genome Analysis ToolKit and potential functions of variants were annotated using ANNOVAR v. 2016Feb01 (Wang *et al.* 2010) and SeattleSequation 138.

Quality control was conducted at both variant and sample levels, as described previously (Wang *et al.* 2015; Dueker *et al.* 2016). Within each individual sample, variants with a depth <4 or Phred-Like score <100 were set as missing. Variants with VQSLOD <−4 and variants with call rate <75% were removed from further analysis. Individuals with low concordance (<95%) between the sequencing data and available genotype data were removed (*n* = 3). Additionally, individuals missing LVM measures (*n* = 9) and/or covariate values (*n* = 1) were removed. For the remaining family study samples, pedigree structure was confirmed using the Graphical Relationship Representation software v. 1.2.1.41. Mendelian error checking was performed, and Mendelian errors were set to missing for all the variants called using PLATO v. 0.84 (Grady *et al.* 2010).

### Replication sample genotyping and quality control

#### Exome Array:

NOMAS participants and our 12 replication families were genotyped using the Illumina HumanExome-24v1_B Beadchip, at the Hussman Institute for Human Genomics in the Center for Genome Technology (Miami, FL). Our Exome Array included custom exonic variants selected on the basis of sequencing data obtained in the discovery family data set. Details of the variant selection have been described previously (Wang *et al.* 2015). A total of 4128 single nucleotide variants (SNVs) within our 12p11 region were available for quality control analyses.

Of the 659 NOMAS participants and 150 replication family members genotyped on the Exome Array, 99.8% had a genotype call rate >98%. A subset of NOMAS participants genotyped on the Exome Array also had Affymetrix 6.0 whole-genome genotype data available and all participants had high concordance with this additional data set (≥96%). We removed seven NOMAS individuals and five replication family members due to unexpected duplication or relatedness, gender discrepancy, and low call rate (<98%). For our current study, individuals missing LVM measures and/or covariate values were removed (*n* = 38 NOMAS participants; *n* = 2 replication family members), leaving us with a final sample of 618 Dominican NOMAS participants and 143 replication family members. At the variant level, we removed SNVs with call rate <95% (*n* = 3 in NOMAS; *n* = 7 in the replication families) and monomorphic SNVs, leaving us with 1419 exonic rare single nucleotide variants (RVs) in our region for Exome Array analysis in NOMAS and 842 exonic RVs in the replication families. Mendelian error checking was performed in the replication families and Mendelian errors were set to missing for all the variants called using PLATO v. 0.84 (Grady *et al.* 2010).

#### Affymetrix 6.0 genotyping chip:

In addition to Exome Array data, many of our NOMAS participants also had Affymetrix 6.0 whole-genome genotyping data available. These data were used for our common single nucleotide variant (CV) analyses since only 10.5% of CVs identified in the discovery families were available on the Exome Array (*n* = 574). Details of our genotyping and QC have been reported previously (Della-Morte *et al.* 2011). These data were imputed using the 1000 Genomes phase 1, version 3 reference panel with IMPUTE2 v.2.2.2 (Howie *et al.* 2009). Variants with INFO ≤0.4 were removed from analyses.

### Statistical methods

#### Family-based discovery analyses:

As in previous analyses, LVM was natural log transformed and multiplied by 10 to ensure it was normally distributed and properly scaled for analyses in SOLAR 6.6.2 (Wang *et al.* 2009; Della-Morte *et al.* 2011). Common and rare SNVs were defined based on frequencies from Dominican NOMAS participants, as described previously (Wang *et al.* 2015). SNVs were classified as common if they had MAF ≥5% and rare if they had MAF <5% or could not be imputed efficiently (INFO ≤0.4) in NOMAS Dominicans. Analyses in the families were performed using the sequencing data. Single-variant analyses were performed for CVs using the Quantitative Transmission-Disequilibrium Test (QTDT), implemented in SOLAR. Adjustment was made for sex, BMI, systolic blood pressure, and smoking status. Covariates were identified using a polygenic screen implemented in SOLAR, with covariates having *P* < 0.1 included in analyses. CVs with *P* < 9.13 × 10^−6^ were considered significant based on a Bonferroni correction of 5477 tests.

To evaluate the contribution of our most strongly associated CV to our linkage results, linkage analyses were performed in our combined sample of sequenced and replication families in SOLAR following our previously detailed protocol (Wang *et al.* 2009). Briefly, linkage analyses were run with and without CV genotype as a covariate and a likelihood ratio test was performed to determine if the LOD score significantly decreased after conditioning on the CV.

Gene-based analyses were performed for RVs using the Family SNP-set (Sequence) Kernel Association Test (Fam-SKAT) v. 1.8 (Chen *et al.* 2013), adjusting for the same covariates included in our single-variant analyses. We employed two different gene-based analyses based on annotation from ANNOVAR v. 2016Feb01 (Wang *et al.* 2010) and SeattleSequation 138: exonic RVs (UTR3, UTR5, synonymous, missense, nonsense, or splice-site variants) and a subset of nonsynonymous RVs only (missense, nonsense, or splice-site variants). These analyses were restricted to genes with ≥2 polymorphic variants and a *P* < 1.83 × 10^−4^ was considered significant based on a Bonferroni correction of 273 tests (181 exonic RV genes and 92 nonsynonymous RV genes).

Using SAS v. 9.3, we computed the residual LVM value after adjusting for the associated risk factors to better visualize the distribution of RVs in relation to LVM.

#### Replication analyses:

LVM was natural log transformed and multiplied by 10 to ensure a normal distribution. We additionally removed outliers falling 3 SD above or below the mean LVM value. Single-variant CV analyses were performed in the NOMAS sample using linear regression, implemented in PLINK v. 1.7. Variants were coded additively. Replication was defined as CVs with *P* < 0.05 and having the same direction of effect. Affy 6.0 genotyping data were used for these analyses.

Gene-based RV association analyses using Exome Array data were performed using SKAT-O v. 1.1.2 in the NOMAS sample and Fam-SKAT v. 1.8 in the replication family sample. These analyses were performed using the same two filtering algorithms employed in the discovery analyses; all exonic RVs and then a subset of nonsynonymous RVs only. Analyses were restricted to genes with ≥2 polymorphic variants. Replication was defined as genes with *P* < 0.05. All association analyses were adjusted for the same covariates included in our discovery analyses. NOMAS analyses were additionally adjusted for factors that were associated with LVM (*P* < 0.1) in this sample: diabetes, time between LVM measurement and baseline patient assessment, waist hip ratio, and the first principal component obtained via principal components analyses implemented in Eigenstrat to account for population substructure. Details of our Eigenstrat analysis and resulting principal components can be found in Figure S1 and File S1.

### Data availability

The data that support the findings of this study are currently being uploaded to dbGaP.

## Results

### Participant and SNV characteristics

A total of 180 individuals in 10 Dominican families were included in our discovery analyses. Characteristics of these 10 families are summarized in Table 1. Family size ranged from 10 to 35 individuals and family-specific LOD scores for the 12p11 region ranged from 0.14 to 0.94. LVM residual values ranged from −5.62 to 6.89. Mean age was between 40 and 54 yr and mean BMI was similar across the 10 families. Variability was seen with respect to percent of participants with diabetes and hypertension in each family (Table 1).

Within the 10 families, sequencing identified 5473 CVs and 10,167 RVs. Among RVs, 24.6% (*n* = 2503) were novel and 22.8% (*n* = 2318) were classified as exonic. A total of 20.2% of exonic RVs (*n* = 468 variants) were either missense, nonsense, or splice-site variants (Table 2). In the NOMAS sample, 96.5% of the CVs and 44.4% of the exonic RVs identified through sequencing were available for replication analyses. An additional 572 exonic RVs on the Exome Array were polymorphic in the NOMAS sample but were monomorphic in the discovery family sample and were included in gene-based replication analyses. In the replication families, 29.6% of the exonic RVs identified through sequencing were available for analysis in addition to 153 exonic RVs that were on the Exome Array and polymorphic in the replication families but monomorphic in the discovery family sample.

**Table 2:** Polymorphic variants identified by resequencing of chr12p11 region in 10 Dominican families

		MAF*^a^*		
Type of Variant*^b^*	Total	Novel	<5%	≥5%
Missense	608	85	372	151
Nonsense	7	1	5	1
Synonymous	598	67	316	215
Splice site	6	1	4	1
UTR 3′ or UTR 5′	2042	374	995	673
ncRNA exonic	127	32	66	29
Intronic	11,380	1839	5627	3914
ncRNA intronic	251	43	116	92
Upstream or downstream	315	61	158	96
ncRNA Other	8	0	5	3
Intergenic	298	0	0	298
Total	15,640	2503	7664	5473

a MAF based on NOMAS DR frequencies.

b Function based on ANNOVAR(2016Feb01) annotation and dbSNP138/GVS 138 annotation.

### CV association results

**Figure 1:**
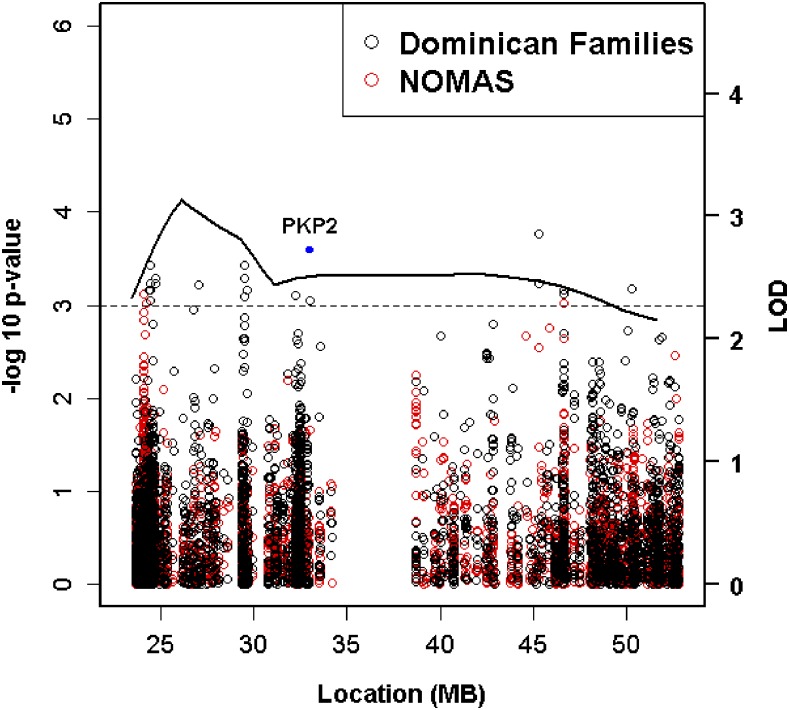
Peak-wide common variant association results for the chr12p11 region. The multipoint LOD score over the region is shown as a solid line. Red circles represent Quantitative Transmission-Disequilibrium *P*-values in the Dominican families and black circles represent linear regression *P*-values in NOMAS. The blue dot above the multipoint LOD score line indicates the position of *PKP2*. The dashed line indicates suggestive association at *P* < 0.001.

Results from the CV analyses are shown in Figure 1 and our most strongly associated variants with suggestive evidence for replication are listed in Table 3. While no CV reached peak-wide significance (*P* < 9.2 × 10^−6^), rs1046116 achieved a *P*-value of 9.0 × 10^−4^ and showed evidence for replication in NOMAS (*P* = 0.03). This variant was associated with decreased LVM and is a missense variant located within *PKP2*. To investigate this association further, we tested rs1046116 for association with LVM in our replication families and found no evidence for replication (*P* = 0.46). To evaluate the contribution of rs1046116 to our linkage signal, we performed linkage analyses in our combined discovery and replication family sample and observed a change in LOD score from 9.09 to 8.30 (*P* < 0.0001) with rs1046116 adjustment, indicating that this CV accounted for some of the linkage signal at the 12p11 QTL. Eight additional variants showed suggestive evidence for association in both the families and NOMAS (*P* < 0.05). The minor allele of these variants, except for rs11168985 (located in *CPNE8*), was associated with increased LVM.

**Table 3:** Common variants with *P* < 0.05 in the discovery families and evidence for replication in NOMAS

				Discovery Families			NOMAS			
Gene	SNP	BP	Effect Allele/Reference Allele	EAF	Direction of Effect*^a^*	*P*-value*^b^*	MAF	Direction of Effect*^a^*	*P*-value*^b^*	Function
*PKP2*	rs1046116	33,021,934	G/A	0.15	−	**9.04 × 10^−4^**	0.20	−	**0.03**	Missense
*ERGIC2*	rs1035607	29,509,513	C/A	0.39	+	**4.72 × 10^−3^**	0.42	+	**0.05**	Intronic
*OR10AD1*	rs11168459	48,596,241	G/A	0.26	+	**0.01**	0.24	+	**0.02**	Missense
*SLC38A4*	rs2191162	47,197,648	A/G	0.27	+	**0.01**	0.34	+	**0.03**	Intronic
*VDR*	rs731236	48,238,757	G/A	0.29	+	**0.02**	0.34	+	**0.03**	Synonymous
*ANO6*	rs74081827	45,833,755	A/G	0.08	+	**0.03**	0.09	+	**1.99 × 10^−3^**	3′ UTR
*TSPAN11*	rs35989439	31,145,916	T/A	0.21	+	**0.03**	0.13	+	**0.01**	3′ UTR
*CPNE8*	rs11168985	39,045,983	A/C	0.25	−	**0.04**	0.22	−	**0.01**	Downstream
*RPAP3*	rs7311790	48,061,435	A/G	0.04	+	**0.04**	0.07	+	**0.02**	Intronic

a + indicates effect allele associated with increased LVM; − indicates effect allele associated with decreased LVM.

b QTDT *P*-value for families, linear regression *P*-value for NOMAS. Bold typeface indicates p-value <0.05.

### RV association results

Gene-based RV analyses were performed using two filtering algorithms; first, analyzing all exonic RVs (*n* = 181 genes) and second, restricting analyses to nonsynonymous RVs (*n* = 92 genes). A total of 54 genes showed suggestive evidence for association in the discovery families under at least one filtering algorithm (*P* < 0.05). Results for these genes are shown in Table S2. Among these top genes, we observed at least suggestive evidence for replication of five genes in NOMAS and three genes in the replication families (*P* < 0.10). Summary results for these genes are shown in Table 4. In exonic RV analyses, *NELL2* was our most strongly associated gene (*P* = 2.2 × 10^−4^), although this finding did not replicate in NOMAS (*P* = 0.53) or the replication families (*P* = 0.18) (Table S2). While no gene achieved replication in both replication samples, our most strongly associated gene with evidence for replication in NOMAS was *ALG10B* (*P* = 0.02 in the discovery families; *P* = 0.04 in NOMAS; *P* = 0.86 in the replication families). When analyses were restricted to nonsynonymous RVs, this association became slightly attenuated in the discovery families (*P* = 0.06), and was even stronger in NOMAS (*P* = 0.03). *GXYLT1* was our most strongly associated gene with evidence for replication in the replication families (*P* = 0.04 in the discovery families; *P* = 0.004 in the replication families; *P* = 0.36 in NOMAS).

**Table 4:** Gene-based association results for rare variant analysis in the chr12p11 region, for genes with *P* < 0.05 in the Dominican families and *P* < 0.10 in the replication families or NOMAS

		Exonic RVs						Nonsynonymous RVs					
		Discovery Families		Replication Families		NOMAS		Discovery Families		Replication Families		NOMAS	
Gene	MB Start	#SNVs	Pval	#SNVs	Pval	#SNVs	Pval	#SNVs	Pval	#SNVs	Pval	#SNVs	Pval
*NELL2*	44.90	24	2.2 × 10^−4^	5	0.18	8	0.53	7	0.006	2	0.02	5	1.00
*PKP2*	32.94	16	4.68 × 10^−3^	6	0.98	14	0.13	6	0.05	6	0.98	11	0.05
*ALG10B*	38.71	42	0.02	9	0.86	22	0.04	7	0.06	2	0.81	7	0.03
*FAM186A*	50.72	26	0.02	15	0.16	21	0.04	18	0.06	12	0.20	18	0.12
*ZNF641*	48.73	17	0.03	–	–	8	0.11	3	0.36	–	–	5	0.15
*PPFIBP1*	27.67	23	0.05	7	0.33	12	2.54 × 10^−3^	6	0.12	5	0.39	9	9.53 × 10^−3^
*SLC2A13*	40.14	19	0.06	7	0.39	9	0.16	2	0.02	3	0.53	3	0.04
*GXYLT1*	42.48	24	0.04	2	4.27 × 10^−3^	9	0.36	–	–	–	–	3	0.85
*SLC38A4*	47.16	21	0.02	6	0.07	12	0.89	3	0.29	–	–	5	0.88

**Figure 2:**
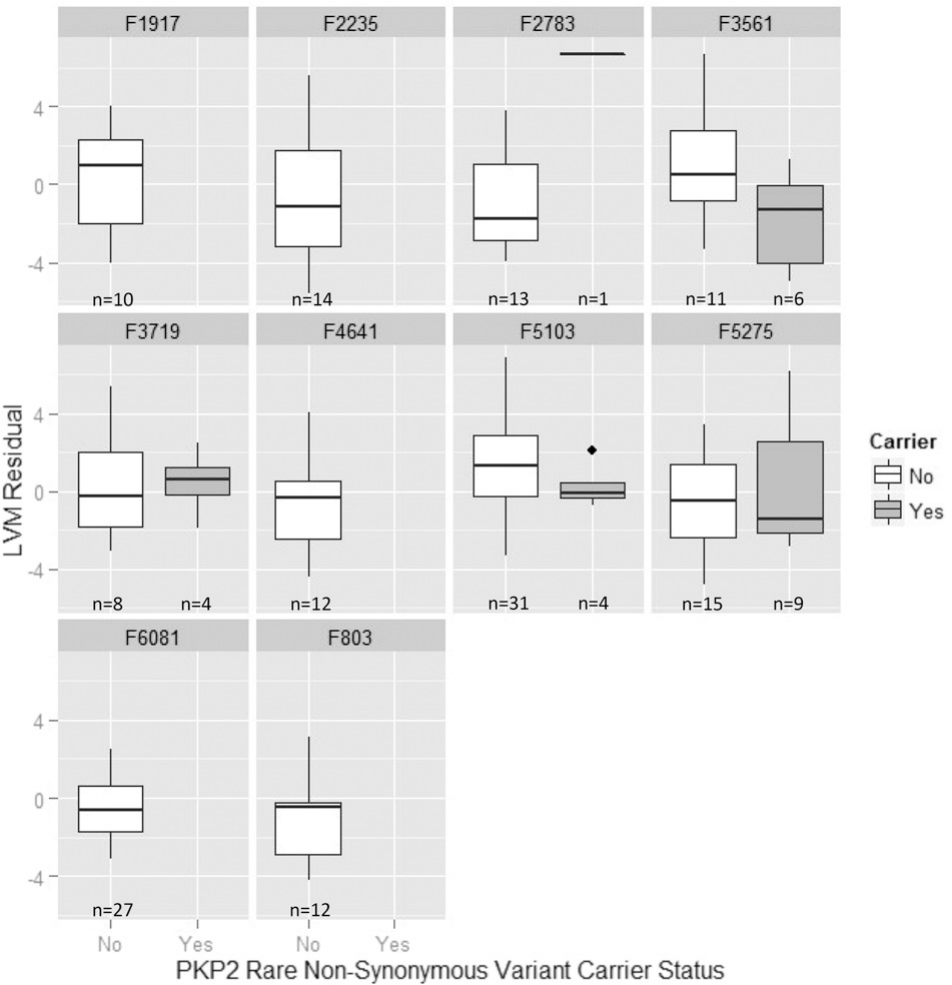
Box and whisker plots showing the distribution of LVM residual by *PKP2* nonsynonymous rare variant carrier status, stratified by family. LVM residuals were calculated by adjusting for sex, body mass index (BMI), systolic blood pressure, and smoking status. Carriers of a *PKP2* nonsynonymous rare variant are shown in gray and noncarriers are shown in white.

**Figure 3:**
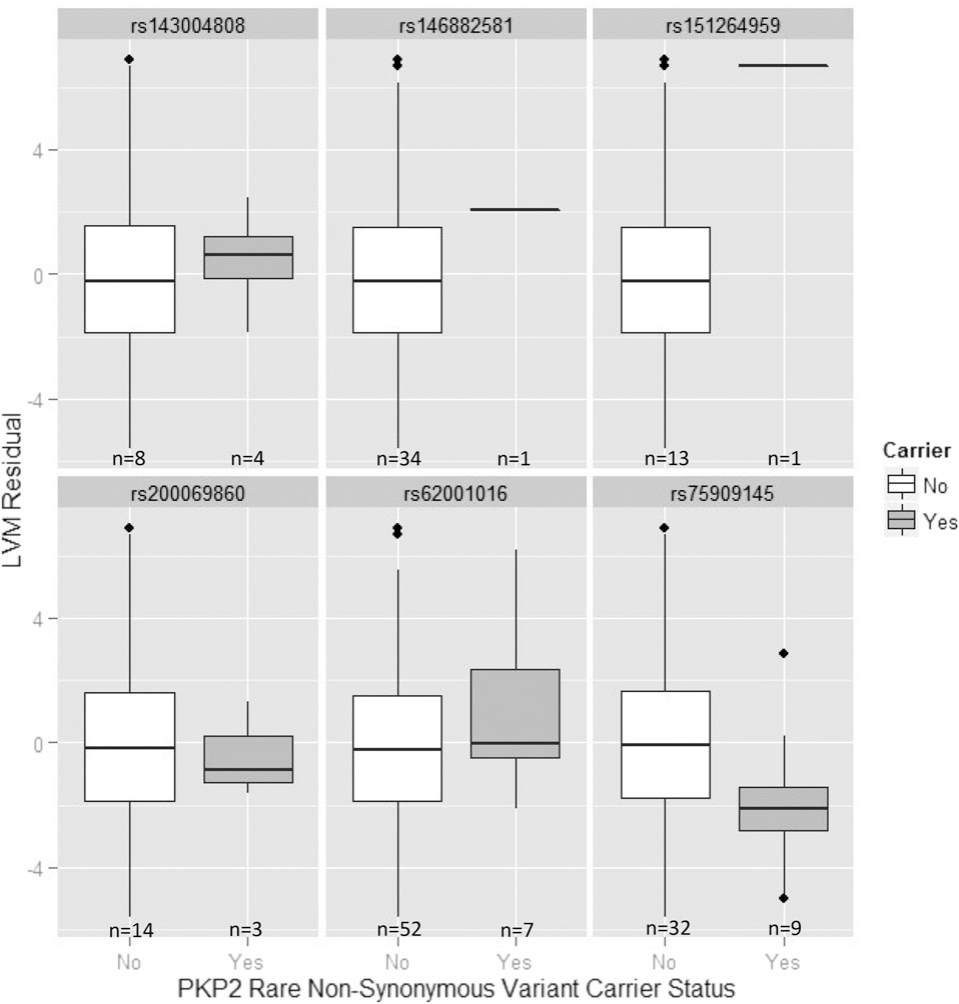
Box and whisker plots showing the distribution of LVM residual within the 10 sequenced families by *PKP2* nonsynonymous rare variant carrier status, stratified by variant. LVM residuals were calculated by adjusting for sex, body mass index (BMI), systolic blood pressure, and smoking status. Carriers of a rare allele of a *PKP2* variant are shown in gray and noncarriers are shown in white.

In nonsynonymous RV analyses, *IFLTD1* showed the strongest evidence for association in the discovery families (*P* = 0.003). However, since no variants met our inclusion criteria in NOMAS or the replication families, replication was not possible. *NELL2* was our second most strongly associated gene (*P* = 0.006), and replicated in the replication families (*P* = 0.02) but not NOMAS (*P* = 1.00) (Table S2). Two genes, *PKP2* and *SLC2A13*, were moderately associated in the discovery families and showed at least suggestive evidence for replication in NOMAS (*P* < 0.10), although they did not replicate in the replication families. Details of the nonsynonymous RVs in *PKP2*, *SLC2A13*, and *NELL2* are shown in Table 5. Within the discovery families, six nonsynonymous variants in *PKP2* were observed, all of which were missense variants. These variants were carried by individuals within five families: F2783, F3561, F3719, F5103, and F5275. In families F2783 and F3719, carriers of a rare *PKP2* allele had higher average LVM residual compared to noncarriers. In the remaining families, carriers of a rare *PKP2* allele had lower average LVM residual compared to noncarriers (Figure 2). When examining each missense variant individually, we observed that carriers of the rare allele of rs143004808, rs146882581, rs151264959, and rs62001016 had higher average LVM residual compared to noncarriers. In contrast, carriers of the rare allele of rs200069860 and rs75909145 had lower average LVM residual compared to noncarriers (Figure 3).

**Table 5:** Annotations for nonsynonymous RVs in *PKP2*, *SLC2A13*, and *NELL2*

					MAF							
Gene	Variant	Position*^a^*	Minor/Major Allele	Discovery Families with Variant	Discovery Families	Replication Families	NOMAS	Function	CADD Score	GERP Score	Amino Acids	PolyPhen*^b^*
*PKP2*	rs200069860	3,3030,850	A/T	F3561	0.008	–	–	Missense	18.04	5.37	GLY/CYS	Probably-damaging
*PKP2*	rs151264959	32,949,047	T/C	F2783	0.003	0.007	7.6 × 10^−4^	Missense	17.76	5.06	ASP/ASN	Probably-damaging
*PKP2*	rs146882581	32,994,073	A/G	F5103	0.003	0.007	0.008	Missense	3.33	2.01	THR/MET	Benign
*PKP2*	rs62001016	33,031,023	A/G	F5103, F5275	0.02	0.01	0.01	Missense	1.72	3.84	ALA/VAL	Benign
*PKP2*	rs75909145	33,049,457	A/C	F3561, F5275	0.03	–	–	Missense	14.02	3.17	SER/ILE	Benign
*PKP2*	rs143004808	33,049,590	T/C	F3719	0.008	–	0.005	Missense	33.00	4.07	ASP/ASN	Probably-damaging
*PKP2*	rs112592855	32,949,140	C/T	–	–	0.003	0.002	Missense	12.39	5.06	THR/ALA	Benign
*PKP2*	rs140852019	32,974,348	C/T	–	–	–	7.6 × 10^−4^	Missense	12.98	2.55	ASN/SER	Benign
*PKP2*	rs139159464	32,996,248	T/C	–	–	–	7.6 × 10^−4^	Splice site	6.18	1.10	–	Unknown
*PKP2*	rs201803918	33,030,840	A/G	–	–	–	7.6 × 10^−4^	Missense	11.25	5.37	ALA/VAL	Benign
*PKP2*	rs149542398	33,031,888	T/C	–	–	–	7.6 × 10^−4^	Missense	15.64	0.71	ARG/HIS	Benign
*PKP2*	12_32975421	32,975,421	A/G	–	–	–	0.01	Nonsense	39.00	4.16	ARG/Stop	Unknown
*PKP2*	12_33021968	33,021,968	A/G	–	–	–	0.005	Nonsense	38.00	2.05	ARG/Stop	Unknown
*PKP2*	rs146102241	32,977,026	T/C	–	–	0.007	–	Missense	23.60	5.32	VAL/ILE	Probably-damaging
*PKP2*	rs139734328	32,949,101	T/G	–	–	0.007	–	Missense	17.80	5.06	ARG/SER	Benign
*SLC2A13*	rs139518863	40,499,594	T/C	F3719	0.005	0.020	0.01	Missense	19.66	4.00	SER/ASN	Possibly-damaging
*SLC2A13*	rs186341127	40,499,132	A/G	F5103	0.008	0.010	0.01	Missense	7.55	1.54	ALA/VAL	Benign
*SLC2A13*	rs146020551	40,265,659	G/A	–	–	0.02	0.002	Missense	12.44	4.57	VAL/ALA	Benign
*NELL2*	rs367712742	44,902,736	C/T	F5103	0.01	–	–	Missense	11.99	5.25	GLN/ARG	Possibly-damaging
*NELL2*	12_45059356	45,059,356	T/C	F6081	0.003	–	–	Missense	9.88	4.38	ARG/HIS	Probably-damaging
*NELL2*	rs144730385	45,105,152	T/C	F3719	0.01	–	0.002	Missense	6.04	1.40	SER/ASN	Benign
*NELL2*	rs17574839	45,108,480	C/T	F5103, F5275	0.03	–	–	Missense	9.57	4.62	ASN/ASP	Benign
*NELL2*	rs201652982	45,171,085	T/C	F6081	0.003	–	–	Missense	32.00	5.62	ASP/ASN	Probably-damaging
*NELL2*	rs372522341	45,269,034	C/T	F6081	0.006	–	–	Missense	16.52	5.14	ASN/SER	Possibly-damaging
*NELL2*	rs2658973	45,269,640	T/C	F2235, F6081	0.008	0.05	0.02	Missense	13.58	3.08	VAL/ILE	Benign
*NELL2*	rs138454729	45,059,310	C/G	–	–	0.003	0.0008	Missense	18.80	4.39	ILE/MET	Possibly-damaging
*NELL2*	12_44926372	44,926,372	A/G	–	–	–	0.0008	Missense	27.50	5.72	SER/LEU	Probably-damaging
*NELL2*	rs146936717	44,915,791	T/G	–	–	–	0.002	Missense	17.79	4.71	ARG/SER	Probably-damaging

a Position based on hg19.

b PPH HumanDiv.

Since our most strongly associated CV was a missense variant in *PKP2*, we performed an additional gene-based analysis on all nonsynonymous variants in *PKP2* (common and rare) which resulted in a slightly stronger association in the discovery families (*P* = 0.04, seven variants) and no association in NOMAS (*P* = 0.41, 12 variants) or the replication families (*P* = 0.98, seven variants).

### Candidate variant and gene association results

We additionally tested whether candidate CVs identified in our previous study of LVM (Della-Morte *et al.* 2011) (*n* = 33 CVs in 14 genes), a study which primarily investigated CV associations with LVM in all Caribbean Hispanics, were associated in our current study which focused specifically on Dominicans. Association results for these CVs are summarized in Table S3. Eleven of the previously reported CVs, located in *SLC38A1*, *BICD1*, *RACGAP1*, *ARID2*, *FAR2*, and *c12orf68*, were found in our discovery families. These CVs included three intronic variants in *SLC38A1* which showed suggestive evidence for association in the discovery families and NOMAS, although the direction of effect differed (rs11183394, rs6582621, rs7133522). One other CV in *SLC38A1*, rs7956629, was moderately associated in the discovery families (*P* = 0.02), but did not replicate in NOMAS (*P* = 0.29). No other CVs were associated with LVM in the families.

We also performed gene-based RV analyses on 14 genes containing the previously reported candidate CVs. Results are summarized in Table S4. Exonic RV analyses identified *PDZRN4* (*P* = 0.03) and *ARID2* (*P* = 0.04) to be moderately associated in the discovery families, although neither replicated in NOMAS or the replication families (*P* > 0.05). One additional gene, *c12orf68*, was moderately associated in the discovery families (*P* = 0.03); however, replication was not possible as no variants met our inclusion criteria in either replication sample. In nonsynonymous RV analyses, *SLC2A13* was the most strongly associated gene and was a top gene in our peak-wide nonsynonymous RV analyses.

## Discussion

Building upon our previous studies which identified a region on 12p11 to be linked with LVM in Dominicans, we performed targeted resequencing in 10 extended families with evidence for linkage to refine the region and identify LVM susceptibility genes. Through these resequencing efforts, we found suggestive evidence for both common and rare variants within 12p11 to influence LVM in Dominican families. Common variant analyses revealed rs1046116, a missense variant in *PKP2*, to be the most strongly associated variant with evidence for replication in our population-based sample of Dominicans. When investigating this association further by testing rs1046116 for association in our 12 additional families with suggestive evidence for linkage to the region, we observed no association; however, these families generally showed weaker evidence for linkage to this region than the 10 families sequenced for our discovery analyses. Further, although this association could not be replicated in our replication families, linkage analyses in our combined family sample revealed that rs1046116 significantly contributed to the original linkage signal as evidenced by a decrease in LOD score from 9.09 to 8.30 in these families. This variant was associated with decreased LVM, suggesting that the G allele of rs1046116 may be protective against the development of left ventricular hypertrophy.

Interestingly, we also found suggestive evidence that RVs within *PKP2* influence LVM. In nonsynonymous RV analyses, *PKP2* was one of the most strongly associated genes with suggestive evidence for replication, thus suggesting that both common and rare nonsynonymous variants, particularly missense variants, may influence LVM. Indeed, when a gene-based analysis was performed on all nonsynonymous variants, common and rare, the association between *PKP2* and LVM became slightly stronger in the families, although the association diminished in NOMAS.

To explore the associations underlying *PKP2* further, we performed an in-depth characterization of the individual rare nonsynonymous variants within this gene. Three missense variants were predicted to be probably-damaging by PolyPhen and showed strong evidence of being conserved (rs200069860, rs151264959, rs143004808). Of particular interest was rs143004808, which was observed in F3719 and had a Combined Annotation Dependent Depletion (CADD) score of 33, indicating that this variant is in the top 0.1% of variants with respect to deleteriousness. Two nonsense variants (located at base pair positions 32,975,421 and 33,021,968) also had similar CADD scores and were observed in the NOMAS sample.

Missense variants in *PKP2*, including several of the ones identified in our study, have been previously implicated in cardiac phenotypes (Cerrone *et al.* 2014; Peters 2014; van der Zwaag *et al.* 2009; Saguner *et al.* 2015), making *PKP2* an excellent candidate gene for LVM. *PKP2* encodes plakophilin 2, a member of one of three major protein families found in desmosomes (Getsios *et al.* 2004). Desmosomes are protein structures in cell membranes that maintain adhesion between neighboring cells and serve as anchoring sites for intermediate filaments. They are found in tissues that experience mechanical stress, including the myocardium (Getsios *et al.* 2004; Desai *et al.* 2009). Mutations in *PKP2* are known to play a role in arrythmogenic cardiomyopathies (AC), most notably arrythmogenic right ventricular dysplasia/cardiomyopathy (ARD/C) (Gerull *et al.* 2004; Fernandez-Rosado *et al.* 2015; Groeneweg *et al.* 2015; Trenkwalder *et al.* 2015). Several studies have shown that *PKP2* haploinsufficiency contributes to pathogenesis in AC (Joshi-Mukherjee *et al.* 2008; Hall *et al.* 2009; Kirchner *et al.* 2012; Rasmussen *et al.* 2014).

In characterizing the clinical course of ARD/C, one study found that 9% of ARD/C patients with a *PKP2* mutation had left ventricular dysfunction and patients carrying more than one mutation were three times more likely to have left ventricular dysfunction compared to patients with one mutation (Bhonsale *et al.* 2015). Studies have also provided further evidence suggesting that *PKP2* mutations may impact left ventricular cardiomyopathies (Horimoto *et al.* 2000; Hamid *et al.* 2002; Sen-Chowdhry *et al.* 2007; Saguner *et al.* 2015), including a recent case report that identified a pathogenic *PKP2* deletion in two siblings with left ventricular noncompaction cardiomyopathy (Ramond *et al.* 2017). Together, these studies support the biologic plausibility for a role of *PKP2* in LVM.

In addition to *PKP2*, our analyses revealed nonsynonymous variants within *SLC2A13* to be suggestively associated in both our discovery families and the NOMAS sample. Included in our nonsynonymous analyses were two missense variants observed in both the families and NOMAS (rs139518863 and rs186341127), as well as one missense variant observed in NOMAS only (rs146020551). Rs139518863 has strong evidence of being evolutionarily conserved (Genomic Evolutionary Rate Profiling (GERP) score = 4), is predicted to be possibly-damaging according to PolyPhen, and has a CADD score of 19.66 indicating that this variant is in the top 10–1% of all variants with respect to deleteriousness. Similar characteristics were observed for rs146020551. Interestingly, our previous association study found CVs in *SLC2A13* to be associated with LVM in Caribbean Hispanics (Della-Morte *et al.* 2011). These findings, in combination with results from our current study, suggest that variants in *SLC2A13* may be involved in LVM in Dominicans and, more broadly, Caribbean Hispanics.

Additional candidate genes identified through our resequencing study were *GXYLT1* and *NELL2. GXYLT1* was identified in our exonic RV analyses and encodes an enzyme that adds xylose to *O*-glucose residues bound to epidermal growth factor repeats of Notch proteins (Sethi *et al.* 2010). Notch signaling plays a role in cardiac development and mutations within Notch signaling genes have been associated with cardiac structural abnormalities including left ventricular outflow tract abnormalities, making *GXYLT1* an excellent candidate gene for LVM (Nemir and Pedrazzini 2008; Padang *et al.* 2012; Penton *et al.* 2012). *NELL2* was one of our most strongly associated genes in RV analyses and encodes the neural epidermal growth factor-like 2 protein which is largely expressed in brain but is also expressed in hematopoietic cells (Luce and Burrows 1999). Its role in LVM is unknown.

When evaluating the results from our study, there are several limitations which should be noted. First, only a subset of families was sequenced; however, these were the families with the strongest evidence for linkage to the 12p11 region. Second, sequencing was performed primarily on exons, therefore missing noncoding regions affecting LVM. However, for candidate genes previously implicated in LVM, sequencing beyond the exons was performed to allow for identification of noncoding variants. Third, the RV replication analyses were performed using Exome Array data, thereby limiting our analyses to only those variants included on the Exome Array. However, variants on the Exome Array were selected to be functional (primarily missense variants) and had to be observed at least three times in at least two people (http://genome.sph.umich.edu/wiki/Exome_Chip_Design), and we added custom content within the 12p11 region to our Exome Array. Fourth, our study was limited to individuals from the Dominican Republic and may not be generalizable to other Hispanic or non-Hispanic populations. Fifth, due to our sample sizes, we had limited power to detect RVs in our study.

In conclusion, our current targeted resequencing study, in combination with our previous studies, shows evidence of a role for both common and rare variants within the 12p11 region in LVM pathogenesis, particularly missense variants within *PKP2*. Functional studies are needed to elucidate the mechanism underlying the association of the implicated genes with LVM.

## 

## Supplementary Material

Supplemental material is available online at www.g3journal.org/lookup/suppl/doi:10.1534/g3.117.300358/-/DC1.

## References

[bib1] Arnett D. K. Li N. Tang W. Rao D. C. Devereux R. B., 2009 Genome-wide association study identifies single-nucleotide polymorphism in KCNB1 associated with left ventricular mass in humans: the HyperGEN Study. BMC Med. Genet. 10: 43. 19454037 10.1186/1471-2350-10-43 PMC2692849

[bib2] Assimes T. L. Narasimhan B. Seto T. B. Yoon S. Curb J. D., 2007 Heritability of left ventricular mass in Japanese families living in Hawaii: the SAPPHIRe Study. J. Hypertens. 25: 985–992. 17414662 10.1097/HJH.0b013e32809bd740

[bib3] Bhonsale A. Groeneweg J. A. James C. A. Dooijes D. Tichnell C., 2015 Impact of genotype on clinical course in arrhythmogenic right ventricular dysplasia/cardiomyopathy-associated mutation carriers. Eur. Heart J. 36: 847–855. 25616645 10.1093/eurheartj/ehu509

[bib4] Cerrone M. Lin X. Zhang M. Agullo-Pascual E. Pfenniger A., 2014 Missense mutations in plakophilin-2 cause sodium current deficit and associate with a Brugada syndrome phenotype. Circulation 129: 1092–1103. 24352520 10.1161/CIRCULATIONAHA.113.003077 PMC3954430

[bib5] Chen H. Meigs J. B. Dupuis J., 2013 Sequence kernel association test for quantitative traits in family samples. Genet. Epidemiol. 37: 196–204. 23280576 10.1002/gepi.21703 PMC3642218

[bib6] Della-Morte D. Beecham A. Rundek T. Wang L. McClendon M. S., 2011 A follow-up study for left ventricular mass on chromosome 12p11 identifies potential candidate genes. BMC Med. Genet. 12: 100. 21791083 10.1186/1471-2350-12-100 PMC3199748

[bib7] Desai B. V. Harmon R. M. Green K. J., 2009 Desmosomes at a glance. J. Cell Sci. 122: 4401–4407. 19955337 10.1242/jcs.037457 PMC2787455

[bib8] de Simone G. Tang W. Devereux R. B. Hunt S. C. Kitzman D. W., 2007 Assessment of the interaction of heritability of volume load and left ventricular mass: the HyperGEN offspring study. J. Hypertens. 25: 1397–1402. 17563561 10.1097/HJH.0b013e328126851e

[bib9] Devereux R. B. Alonso D. R. Lutas E. M. Gottlieb G. J. Campo E., 1986 Echocardiographic assessment of left ventricular hypertrophy: comparison to necropsy findings. Am. J. Cardiol. 57: 450–458. 2936235 10.1016/0002-9149(86)90771-x

[bib10] Devereux R. B. Wachtell K. Gerdts E. Boman K. Nieminen M. S., 2004 Prognostic significance of left ventricular mass change during treatment of hypertension. JAMA 292: 2350–2356. 15547162 10.1001/jama.292.19.2350

[bib11] Di Tullio M. R. Zwas D. R. Sacco R. L. Sciacca R. R. Homma S., 2003 Left ventricular mass and geometry and the risk of ischemic stroke. Stroke 34: 2380–2384. 12958319 10.1161/01.STR.0000089680.77236.60 PMC2812917

[bib12] Dueker N. D. Beecham A. Wang L. Blanton S. H. Guo S., 2016 Rare variants in NOD1 associated with carotid bifurcation intima-media thickness in Dominican Republic families. PLoS One 11: e0167202. 27936005 10.1371/journal.pone.0167202 PMC5147882

[bib13] Elkind M. S. Sciacca R. Boden-Albala B. Rundek T. Paik M. C., 2006 Moderate alcohol consumption reduces risk of ischemic stroke: the Northern Manhattan Study. Stroke 37: 13–19. 16306464 10.1161/01.STR.0000195048.86810.5b

[bib14] Fernandez-Rosado F. Alvarez-Cubero M. J. Entrala-Bernal C. Pino Mdel C. Gomez-Recio M., 2015 Identification by next generation sequencing of a novel PKP2 mutation in arrhythmogenic right ventricular dysplasia. Arch. Med. Res. 46: 170–171. 25707291 10.1016/j.arcmed.2015.02.003

[bib15] Fox E. R. Musani S. K. Barbalic M. Lin H. Yu B., 2013 Genome-wide association study of cardiac structure and systolic function in African Americans: the candidate gene association resource (CARe) study. Circ Cardiovasc Genet 6: 37–46. 23275298 10.1161/CIRCGENETICS.111.962365 PMC3591479

[bib16] Garner C. Lecomte E. Visvikis S. Abergel E. Lathrop M., 2000 Genetic and environmental influences on left ventricular mass. A family study. Hypertension 36: 740–746. 11082137 10.1161/01.hyp.36.5.740

[bib17] Gerull B. Heuser A. Wichter T. Paul M. Basson C. T., 2004 Mutations in the desmosomal protein plakophilin-2 are common in arrhythmogenic right ventricular cardiomyopathy. Nat. Genet. 36: 1162–1164. 15489853 10.1038/ng1461

[bib18] Getsios S. Huen A. C. Green K. J., 2004 Working out the strength and flexibility of desmosomes. Nat. Rev. Mol. Cell Biol. 5: 271–281. 15071552 10.1038/nrm1356

[bib19] Grady B. J. Torstenson E. Dudek S. M. Giles J. Sexton D., 2010 Finding unique filter sets in PLATO: a precursor to efficient interaction analysis in GWAS data. Pac. Symp. Biocomput. 315–326. 19908384 PMC2903053

[bib20] Groeneweg J. A. Bhonsale A. James C. A. te Riele A. S. Dooijes D., 2015 Clinical presentation, long-term follow-up, and outcomes of 1001 arrhythmogenic right ventricular dysplasia/cardiomyopathy patients and family members. Circ Cardiovasc Genet 8: 437–446. 25820315 10.1161/CIRCGENETICS.114.001003

[bib21] Hall C. Li S. Li H. Creason V. Wahl J. K. III, 2009 Arrhythmogenic right ventricular cardiomyopathy plakophilin-2 mutations disrupt desmosome assembly and stability. Cell Commun. Adhes. 16: 15–27. 19533476 10.1080/15419060903009329

[bib22] Hamid M. S. Norman M. Quraishi A. Firoozi S. Thaman R., 2002 Prospective evaluation of relatives for familial arrhythmogenic right ventricular cardiomyopathy/dysplasia reveals a need to broaden diagnostic criteria. J. Am. Coll. Cardiol. 40: 1445–1450. 12392835 10.1016/s0735-1097(02)02307-0

[bib23] Harper A. R. Mayosi B. M. Rodriguez A. Rahman T. Hall D., 2013 Common variation neighbouring micro-RNA 22 is associated with increased left ventricular mass. PLoS One 8: e55061. 23372812 10.1371/journal.pone.0055061 PMC3555935

[bib24] Horimoto M. Akino M. Takenaka T. Igarashi K. Inoue H., 2000 Evolution of left ventricular involvement in arrhythmogenic right ventricular cardiomyopathy. Cardiology 93: 197–200. 10965092 10.1159/000007026

[bib25] Howie B. N. Donnelly P. Marchini J., 2009 A flexible and accurate genotype imputation method for the next generation of genome-wide association studies. PLoS Genet. 5: e1000529. 19543373 10.1371/journal.pgen.1000529 PMC2689936

[bib26] Joshi-Mukherjee R. Coombs W. Musa H. Oxford E. Taffet S., 2008 Characterization of the molecular phenotype of two arrhythmogenic right ventricular cardiomyopathy (ARVC)-related plakophilin-2 (PKP2) mutations. Heart Rhythm 5: 1715–1723. 19084810 10.1016/j.hrthm.2008.09.009 PMC2636742

[bib27] Juo S. H. Di Tullio M. R. Lin H. F. Rundek T. Boden-Albala B., 2005 Heritability of left ventricular mass and other morphologic variables in Caribbean Hispanic subjects: the Northern Manhattan Family Study. J. Am. Coll. Cardiol. 46: 735–737. 16098447 10.1016/j.jacc.2005.05.025 PMC2692931

[bib28] Kirchner F. Schuetz A. Boldt L. H. Martens K. Dittmar G., 2012 Molecular insights into arrhythmogenic right ventricular cardiomyopathy caused by plakophilin-2 missense mutations. Circ Cardiovasc Genet 5: 400–411. 22781308 10.1161/CIRCGENETICS.111.961854

[bib29] Levy D. Garrison R. J. Savage D. D. Kannel W. B. Castelli W. P., 1990 Prognostic implications of echocardiographically determined left ventricular mass in the Framingham Heart Study. N. Engl. J. Med. 322: 1561–1566. 2139921 10.1056/NEJM199005313222203

[bib30] Li H. Durbin R., 2010 Fast and accurate long-read alignment with Burrows-Wheeler transform. Bioinformatics 26: 589–595. 20080505 10.1093/bioinformatics/btp698 PMC2828108

[bib31] Luce M. J. Burrows P. D., 1999 The neuronal EGF-related genes NELL1 and NELL2 are expressed in hemopoietic cells and developmentally regulated in the B lineage. Gene 231: 121–126. 10231576 10.1016/s0378-1119(99)00093-1

[bib100] Mozaffarian D. Benjamin E. J. Go A. S. Arnett D. K. Blaha M. J., 2016 Executive summary: heart disease and stroke statistics–2016 update: a report from the American Heart Association. Circulation 133: 447–454. 26811276 10.1161/CIR.0000000000000366

[bib32] Nemir M. Pedrazzini T., 2008 Functional role of Notch signaling in the developing and postnatal heart. J. Mol. Cell. Cardiol. 45: 495–504. 18410944 10.1016/j.yjmcc.2008.02.273

[bib33] Padang R. Bagnall R. D. Semsarian C., 2012 Genetic basis of familial valvular heart disease. Circ Cardiovasc Genet 5: 569–580. 23074336 10.1161/CIRCGENETICS.112.962894

[bib34] Penton A. L. Leonard L. D. Spinner N. B., 2012 Notch signaling in human development and disease. Semin. Cell Dev. Biol. 23: 450–457. 22306179 10.1016/j.semcdb.2012.01.010 PMC3638987

[bib35] Peters S., 2014 Arrhythmogenic cardiomyopathy and provocable brugada ECG in a patient caused by missense mutation in plakophilin-2. Int. J. Cardiol. 173: 317–318. 24681023 10.1016/j.ijcard.2014.03.071

[bib36] Post W. S. Larson M. G. Myers R. H. Galderisi M. Levy D., 1997 Heritability of left ventricular mass: the Framingham Heart Study. Hypertension 30: 1025–1028. 9369250 10.1161/01.hyp.30.5.1025

[bib37] Ramond F. Janin A. Di Filippo S. Chanavat V. Chalabreysse L., 2017 Homozygous PKP2 deletion associated with neonatal left ventricle noncompaction. Clin. Genet. 91: 126–130. 27030002 10.1111/cge.12780

[bib38] Rasmussen T. B. Nissen P. H. Palmfeldt J. Gehmlich K. Dalager S., 2014 Truncating plakophilin-2 mutations in arrhythmogenic cardiomyopathy are associated with protein haploinsufficiency in both myocardium and epidermis. Circ Cardiovasc Genet 7: 230–240. 24704780 10.1161/CIRCGENETICS.113.000338

[bib39] Rodriguez C. J. Lin F. Sacco R. L. Jin Z. Boden-Albala B., 2006 Prognostic implications of left ventricular mass among Hispanics: the Northern Manhattan Study. Hypertension 48: 87–92. 16651457 10.1161/01.HYP.0000223330.03088.58

[bib40] Sacco R. L. Anand K. Lee H. S. Boden-Albala B. Stabler S., 2004 Homocysteine and the risk of ischemic stroke in a triethnic cohort: the Northern Manhattan Study. Stroke 35: 2263–2269. 15345803 10.1161/01.STR.0000142374.33919.92

[bib41] Sacco R. L. Sabala E. A. Rundek T. Boden-Albala B. Stabler S., 2007 Design of a family study among high-risk Caribbean Hispanics: the Northern Manhattan Family Study. Ethn. Dis. 17: 351–357. 17682370 PMC2556080

[bib42] Saguner A. M. Buchmann B. Wyler D. Manka R. Gotschy A., 2015 Arrhythmogenic left ventricular cardiomyopathy: suspected by cardiac magnetic resonance imaging, confirmed by identification of a novel plakophilin-2 variant. Circulation 132: e38–e40. 26260507 10.1161/CIRCULATIONAHA.115.017284

[bib43] Sahn D. J. DeMaria A. Kisslo J. Weyman A., 1978 Recommendations regarding quantitation in M-mode echocardiography: results of a survey of echocardiographic measurements. Circulation 58: 1072–1083. 709763 10.1161/01.cir.58.6.1072

[bib44] Savage D. D. Levy D. Dannenberg A. L. Garrison R. J. Castelli W. P., 1990 Association of echocardiographic left ventricular mass with body size, blood pressure and physical activity (the Framingham Study). Am. J. Cardiol. 65: 371–376. 2137280 10.1016/0002-9149(90)90304-j

[bib45] Sen-Chowdhry S. Syrris P. Ward D. Asimaki A. Sevdalis E., 2007 Clinical and genetic characterization of families with arrhythmogenic right ventricular dysplasia/cardiomyopathy provides novel insights into patterns of disease expression. Circulation 115: 1710–1720. 17372169 10.1161/CIRCULATIONAHA.106.660241

[bib46] Sethi M. K. Buettner F. F. Krylov V. B. Takeuchi H. Nifantiev N. E., 2010 Identification of glycosyltransferase 8 family members as xylosyltransferases acting on O-glucosylated notch epidermal growth factor repeats. J. Biol. Chem. 285: 1582–1586. 19940119 10.1074/jbc.C109.065409 PMC2804315

[bib47] Sharma P. Middelberg R. P. Andrew T. Johnson M. R. Christley H., 2006 Heritability of left ventricular mass in a large cohort of twins. J. Hypertens. 24: 321–324. 16508579 10.1097/01.hjh.0000202815.18083.03

[bib48] Swan L. Birnie D. H. Padmanabhan S. Inglis G. Connell J. M., 2003 The genetic determination of left ventricular mass in healthy adults. Eur. Heart J. 24: 577–582. 12643891 10.1016/s0195-668x(02)00524-9

[bib49] Trenkwalder T. Deisenhofer I. Hadamitzky M. Schunkert H. Reinhard W., 2015 Novel frame-shift mutation in PKP2 associated with arrhythmogenic right ventricular cardiomyopathy: a case report. BMC Med. Genet. 16: 117. 26701096 10.1186/s12881-015-0263-1 PMC4690428

[bib50] van der Zwaag P. A. Jongbloed J. D. van den Berg M. P. van der Smagt J. J. Jongbloed R., 2009 A genetic variants database for arrhythmogenic right ventricular dysplasia/cardiomyopathy. Hum. Mutat. 30: 1278–1283. 19569224 10.1002/humu.21064

[bib51] Vasan R. S. Larson M. G. Aragam J. Wang T. J. Mitchell G. F., 2007 Genome-wide association of echocardiographic dimensions, brachial artery endothelial function and treadmill exercise responses in the Framingham Heart Study. BMC Med. Genet. 8(Suppl. 1): S2. 17903301 10.1186/1471-2350-8-S1-S2 PMC1995617

[bib52] Vasan R. S. Glazer N. L. Felix J. F. Lieb W. Wild P. S., 2009 Genetic variants associated with cardiac structure and function: a meta-analysis and replication of genome-wide association data. JAMA 302: 168–178. 19584346 10.1001/jama.2009.978-a PMC2975567

[bib53] Wang K. Li M. Hakonarson H., 2010 ANNOVAR: functional annotation of genetic variants from next-generation sequencing data. Nucleic Acids Res. 38: e164. 20601685 10.1093/nar/gkq603 PMC2938201

[bib54] Wang L. Beecham A. Di Tullio M. R. Slifer S. Blanton S. H., 2009 Novel quantitative trait locus is mapped to chromosome 12p11 for left ventricular mass in Dominican families: the Family Study of Stroke Risk and Carotid Atherosclerosis. BMC Med. Genet. 10: 74. 19627612 10.1186/1471-2350-10-74 PMC2724377

[bib55] Wang L. Beecham A. Dueker N. Blanton S. H. Rundek T., 2015 Sequencing of candidate genes in Dominican families implicates both rare exonic and common non-exonic variants for carotid intima-media thickness at bifurcation. Hum. Genet. 134: 1127–1138. 26319989 10.1007/s00439-015-1592-z PMC4570583

