# Supplementary material for: Sequencing of Linkage Region on Chromosome 12p11 Identifies PKP2 as a Candidate Gene for Left Ventricular Mass in Dominican Families
Source: G3 (Bethesda). 2017 Dec 29;8(2):659–68. doi: 10.1534/g3.117.300358 (PMC5919734; doi:10.1534/g3.117.300358)
Supplement: Supplementary file 1 [file 659FigureS1.docx]

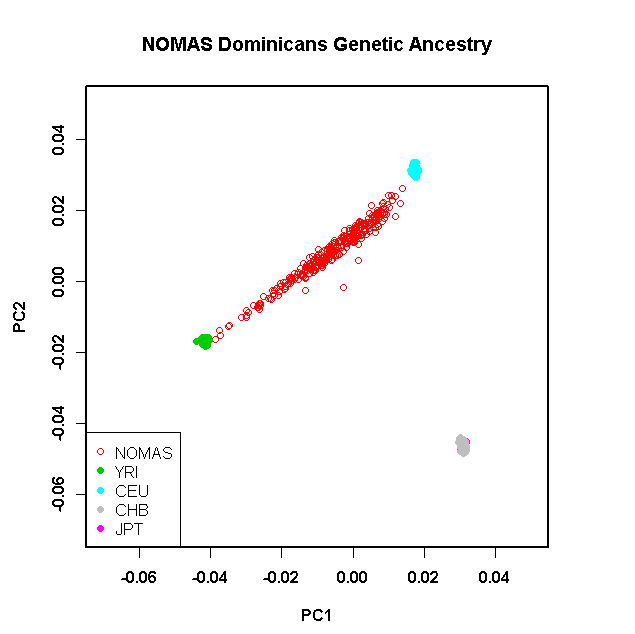


**Supplementary Figure 1. Principal component 1 (PC1) vs. principal component 2 (PC2) for Dominican NOMAS participants and select HapMap3 populations**. YRI=Yorubans from Ibadan, Nigeria, CEU=Utah residents from the CEPH collection, JPT=Japanese from Tokyo, Japan and CHB=Han Chinese from Beijing, China.
