# Supplementary material for: Sequencing of Linkage Region on Chromosome 12p11 Identifies PKP2 as a Candidate Gene for Left Ventricular Mass in Dominican Families
Source: G3 (Bethesda). 2017 Dec 29;8(2):659–68. doi: 10.1534/g3.117.300358 (PMC5919734; doi:10.1534/g3.117.300358)
Supplement: Supplementary file 6 [file 659FileS1.docx]

Supplementary Methods

To characterize the genetic ancestry of our NOMAS replication sample, we performed principal components analysis (PCA) with EIGENSTAT on NOMAS Dominicans and several HapMap3 populations. PCAs were restricted to autosomal variants with call rate > 95% and MAF ≥ 5%. Additionally, variants in pairwise linkage disequilibrium were removed (PLINK option indep-pairwise 100 2 0.8), leaving 49,165 SNPs for analysis. Two main axes of genetic ancestry were present; an axis extending from the HapMap CEU population to the HapMap YRI population and an axis extending from the HapMap CEU population to the HapMap JHP/CHB populations, with our Dominican samples falling along the CEU-YRI axis.
